# Mental Health Disorders in End‐Stage Kidney Disease: A Narrative Review of Clinical Considerations for Integrated Management

**DOI:** 10.1111/hdi.70102

**Published:** 2026-06-16

**Authors:** Kunal Malhotra, Jordan Fangue‐Nelon, Garima Singh

**Affiliations:** ^1^ Division of Nephrology, Department of Medicine Saint Louis University St. Louis Missouri USA; ^2^ Centerstone Nashville Tennessee USA; ^3^ Centerstone St. louis Missouri USA

**Keywords:** CBT, collaborative care, depression, ESKD, mental health

## Abstract

**Introduction:**

End stage kidney disease (ESKD) is associated with a high prevalence of depression and anxiety, arising from both biological factors and the psychosocial burden of chronic dialysis care. Approximately 25%–30% of dialysis patients experience clinically significant depressive symptoms when assessed by structured clinical interview, though reported prevalence varies widely by assessment method. These disorders negatively impact adherence, hospitalization rates, and mortality, with diagnostic challenges arising from overlapping uremic symptoms and reliance on self‐report tools. Biological contributors such as uremia, anemia, chronic inflammation, and polypharmacy, together with social stressors including unemployment, isolation, and financial hardship, exacerbate psychiatric vulnerability.

**Discussion:**

Randomized trials demonstrate that cognitive behavioral therapy (CBT) reduces depressive symptoms, improves quality of life, and enhances treatment adherence, and lessons from oncology and primary care support collaborative care models. However, dialysis centers often lack resources and are constrained by reimbursement policy. This narrative review synthesizes evidence across epidemiology, biopsychosocial mechanisms, pharmacotherapy considerations specific to renal impairment, and non‐pharmacologic interventions, and proposes a roadmap for integrated mental health care in dialysis that clearly distinguishes evidence‐based recommendations from expert opinion. Integrated, multidisciplinary approaches hold promise for enhancing patient well‐being and reducing the burden of mental illness in dialysis care.

## 1 Introduction

Chronic kidney disease (CKD) is defined by persistent evidence of kidney damage or decreased kidney function for at least 3 months, irrespective of the cause. End‐stage kidney disease (ESKD), or kidney failure, occurs when CKD reaches an advanced state (eGFR < 15 mL/min/1.73 m^2^) [1]. People with ESKD require dialysis or kidney transplantation to survive [1]. Once patients reach ESKD, uremic symptoms begin to manifest, including malnutrition, fatigue, anorexia, nausea, vomiting, platelet dysfunction, sexual dysfunction, pericarditis, and neuropathy.

In addition to physical symptoms, ESKD is commonly associated with significant psychological distress, including stress, anxiety, and depression. As physical and mental health decline, patients often find themselves dealing with financial strain, the time demands of ongoing treatment, disruptions to daily routines, and changes in employment. Many experience anxiety driven by concerns about how their illness and treatment affect relationships with family, work, friends, and partners. Depression may follow the initial emotional impact of the diagnosis and is often worsened by dietary restrictions and the broader lifestyle changes required to manage CKD. Together, these challenges contribute to the high burden of mental health disorders among individuals with CKD and ESKD.

In recognition of this burden, the United States Centers for Medicare and Medicaid Services (CMS) mandates that dialysis centers screen for depressive symptoms [2, 3]. The American Kidney Fund advises patients to seek assistance and consult the social worker at their dialysis center [4, 5]. Recommended treatments include CBT and pharmacologic therapy, with CBT specifically cited for its role in interrupting negative thought patterns and restructuring maladaptive behaviors such as missed dialysis sessions or nonadherence to fluid, salt, phosphorus, and medication regimens [6, 7, 8]. CMS further mandates that all US dialysis facilities and kidney transplant centers employ a master's‐trained social worker (MSW) to address patients' psychosocial needs, ensuring completion of psychosocial evaluations, participation in interdisciplinary care planning, provision of case management, and referral to psychologists or psychiatrists when indicated [9, 10, 11]. Despite these requirements, access to structured psychotherapy and integrated psychiatric care remains inconsistent across dialysis settings.

## 2 Methods

This manuscript is a narrative review of the current literature on depression and anxiety in individuals with CKD and ESKD, with particular attention to prevalence, contributing factors, clinical consequences, pharmacologic and non‐pharmacologic management, and implications for integrated care in dialysis settings. A structured literature search was conducted in PubMed/MEDLINE, PsycINFO, and the Cochrane Library for English‐language articles published between January 2000 and October 2025, using combinations of the following key terms: “chronic kidney disease,” “end‐stage kidney disease,” “dialysis,” “hemodialysis,” “depression,” “anxiety,” “mental health,” “cognitive behavioral therapy,” “antidepressant,” “collaborative care,” and “quality of life.” Reference lists of key articles were hand‐searched to identify additional relevant studies. Inclusion was prioritized for randomized controlled trials, systematic reviews, meta‐analyses, large prospective cohorts, clinical practice guidelines, and CMS/MedPAC policy documents. Case reports, conference abstracts without full‐text publication, and non‐English articles were excluded. Articles were selected based on clinical relevance, methodological rigor, and contribution to understanding depression and its management in CKD and ESKD. Findings were synthesized narratively rather than through formal systematic review or meta‐analytic methods. This approach was chosen to allow integration of heterogeneous evidence across epidemiology, mechanism, pharmacology, psychotherapy, and health policy into a clinically oriented synthesis.

## 3 Epidemiology and Diagnostic Challenges

CKD affects approximately one in seven US adults, equating to more than 35.5 million people, most of whom are undiagnosed [12]. CKD is the ninth leading cause of death in the US, causing more deaths annually than breast or prostate cancer. Approximately 40% of individuals with severely reduced kidney function who are not on dialysis are unaware of their condition, and this figure rises to 90% among those with stage 3 CKD [12]. According to the 2024 USRDS Annual Data Report, there were 815,896 dialysis patients in the United States in 2022 [13].

Depression and anxiety are markedly more prevalent among dialysis patients than in the general population or in other chronic disease cohorts. However, reported prevalence estimates vary widely, and this variability has clinical and methodologic implications. Differences arise from the assessment instrument used (symptom‐based self‐report vs. structured clinical interview), from the type of prevalence measured (point vs. period vs. lifetime), from the severity threshold applied, from population characteristics (incident vs. prevalent dialysis, in‐center hemodialysis versus peritoneal dialysis vs. transplant candidates), and from geographic and cultural differences across cohorts [14, 15].

Self‐administered symptom scales such as the Beck Depression Inventory (BDI) and the Patient Health Questionnaire‐9 (PHQ‐9) consistently yield higher prevalence estimates than structured diagnostic interviews because somatic items (fatigue, poor appetite, sleep disturbance) overlap with symptoms of uremia, anemia, and the dialysis procedure itself. When DSM‐5 criteria are applied via structured interview, many somatic symptoms do not meet threshold for a major depressive episode, resulting in markedly lower but more specific estimates. This is highlighted in the pooled meta‐analysis by Palmer et al., reporting an interview‐based prevalence of 22.8% (95% CI, 18.6%–27.6%) in dialysis‐dependent adults versus a self‐report‐based prevalence of 39.3% (95% CI, 36.8%–42.0%) in the same population [15]. A summary of prevalence estimates by assessment method is provided in Table 1. For clinical purposes, we suggest that a prevalence of approximately 20%–25% (structured interview) represents the most defensible figure for clinically significant major depressive disorder in dialysis, while screening‐instrument‐based estimates of 35%–40% more accurately capture the broader burden of clinically meaningful depressive symptoms that warrant further evaluation.

**TABLE 1:** Prevalence of depression in dialysis populations by assessment method.

Assessment method	Instrument/definition	Prevalence	Interpretation
Self‐report symptom scale	BDI ≥ 14; BDI‐II ≥ 16; CES‐D ≥ 16; PHQ‐9 ≥ 10	~35%–40% (pooled); 39.3% (95% CI 36.8–42.0) in stage 5D per Palmer 2013	Captures clinically meaningful depressive symptoms; overestimates MDD due to overlap with uremic/somatic symptoms
Structured clinical interview	SCID/MINI/CIDI for DSM‐IV or DSM‐5 MDD	~20%–25% (pooled); 22.8% (95% CI 18.6–27.6) in stage 5D per Palmer 2013	Most specific estimate of current major depressive disorder; recommended reference standard for clinical diagnosis
PHQ‐9 screening in practice	PHQ‐9 ≥ 10 (CMS threshold for further assessment)	~25%–40%	Point prevalence; positive screen, not a diagnosis
Wide‐range literature estimates	Heterogeneous instruments, thresholds, populations	15%–60%	Reflects methodologic heterogeneity rather than true variation; not recommended as a clinical benchmark
Anxiety disorders (dialysis)	GAD‐7, HADS‐Anxiety, structured interview	~12%–50% (symptoms); ~27%–46% (disorder)	Frequently co‐occurs with depression; often under‐recognized

*Note:* Adapted and synthesized primarily from Palmer et al. [15] and Kondo et al. [14].

Abbreviations: BDI, Beck Depression Inventory; CES‐D, Center for Epidemiologic Studies Depression Scale; CIDI, Composite International Diagnostic Interview; GAD‐7, Generalized Anxiety Disorder 7‐item; HADS, Hospital Anxiety and Depression Scale; MDD, major depressive disorder; MINI, Mini International Neuropsychiatric Interview; PHQ‐9, Patient Health Questionnaire‐9; SCID, Structured Clinical Interview for DSM.

These diagnostic challenges, combined with limited mental health infrastructure in dialysis environments, complicate timely identification and treatment of clinically significant depression and anxiety [6, 14, 15].

## 4 Biopsychosocial Interplay Between ESKD and Mental Health

The elevated prevalence of psychiatric disorders in patients with ESKD reflects a multilevel interaction of biological, psychological, and social determinants. From a biological perspective, uremia and kidney–brain axis dysfunction lead to neuroinflammation, altered neurotransmission, and cognitive decline [16]. These mechanisms contribute to mood disorders independently of psychosocial stress. Chronic inflammation and malnutrition—characterized by elevated cytokines such as IL‐6 and TNF‐α—have been linked to depression and frailty [13]. Fatigue from anemia, present in approximately 90% of dialysis patients, and cognitive impairment from uremic toxin accumulation further worsen emotional health [7, 16].

Psychologically, the chronic and life‐altering nature of ESKD introduces stressors such as fear of death, treatment burden, and existential distress—factors that may not be explicitly reported but significantly impact mood and coping [7]. Unemployment and disability are common in roughly 25%–30% of dialysis patients and contribute to hopelessness, loss of autonomy, and poor treatment adherence [7, 17]. Functional decline and sarcopenia affect nearly half of patients, and over 60% report chronic pain or disturbed sleep—factors known to impair resilience [18, 19]. Comorbid conditions such as diabetes and cardiovascular disease, present in more than 70% of patients, compound the complexity and mental health burden [13].

Social determinants also play a critical role. Approximately 20%–40% of patients report low family or social support, correlating with worse psychological outcomes [17, 20]. Financial strain and cultural or language barriers disproportionately affect underrepresented and immigrant populations, leading to delays in care and underdiagnosed mental health issues [20]. Pandemic‐related disruptions were anticipated to worsen mental health in this vulnerable population; however, one Dutch prospective study (DOMESTICO cohort) found mental health scores were essentially unchanged during COVID‐19 compared to the pre‐pandemic period, suggesting dialysis patients may have greater psychological resilience to social disruption than the general population—possibly because enforced isolation is less novel in the context of chronic dialysis dependence [21]. Body image concerns related to dialysis access sites or weight changes can reduce self‐esteem and foster isolation [17]. Polypharmacy, common in dialysis patients, may contribute to mood alteration via central nervous system side effects [22].

Despite clear evidence of need, access to behavioral health care in dialysis remains limited. Many dialysis units have only one social worker per 75 or more patients, constraining capacity for ongoing psychosocial support [11]. Approximately 20% of ESKD patients have preexisting psychiatric diagnoses or trauma histories that heighten vulnerability to persistent depression, anxiety, and suicide risk [17]. This complex, interrelated matrix of risk factors highlights the urgent need for integrated, multidisciplinary mental health interventions in ESKD care. Tailored strategies addressing both biological mechanisms and structural inequities are essential to improve patient outcomes and quality of life. See Table 2.

**TABLE 2:** Biopsychosocial interplay between ESKD and depression (adapted from refs. [7, 13, 16, 17, 18, 19, 20, 21, 22]).

Contributing factor	Mechanistic pathway	Associated outcomes	Estimated impact/contextual note
Fear of death/existential distress	Psychological distress	Depressive symptoms, treatment withdrawal	Common in ESKD; not directly quantified (qualitative evidence; ref. [7])
Unemployment/disability	Hopelessness, loss of autonomy	Poor adherence, depressive symptoms	25%–30% of dialysis patients (refs [7, 17])
Comorbid illness (DM, CVD)	Symptom burden, polypharmacy	Cognitive decline, reduced survival	> 70% of ESKD patients (USRDS 2024; ref. [13])
Kidney–brain axis dysfunction	Uremic neurotoxicity, BBB disruption	Depression, cognitive impairment	Mechanistic; multiple CKD studies (ref [17])
Low family/social support	Impaired coping, loneliness	MDD, suicidal ideation	20%–40% of dialysis patients (refs [17, 20])
Sarcopenia/functional decline	Fatigue, immobility	Reduced QoL	~50% of dialysis patients (ref [18])
Chronic pain/poor sleep	Dysregulated stress, insomnia	Mood disturbance, reduced resilience	> 60% of dialysis patients (DOPPS; ref. [19])
Malnutrition/inflammation	Elevated IL‐6, TNF‐α	Depressive symptoms, frailty	30%–50% with PEW markers (ref [13])
Uremia	Accumulated neurotoxic metabolites	Apathy, impaired concentration	Universal in ESKD; varies with residual function
Anemia of CKD	Fatigue, reduced cerebral oxygenation	Low motivation, depressed mood	~90% of dialysis patients (ref [13])
Altered body image (fistula, weight)	Stigma, reduced self‐esteem	Social withdrawal, anxiety	~15%–20% in qualitative studies (ref [17])
Polypharmacy	CNS side effects	Emotional blunting, poor adherence	> 60% take ≥ 5 medications (ref [22])
Financial strain	Economic hardship, medication access	Psychological stress, depression	Especially in underinsured/minority groups (ref [20])
Cultural/language barriers	Inequitable MH care access	Underdiagnosed depression	Non‐English speakers, immigrant groups (ref [20])
Limited psychiatric services	No integrated BH in dialysis units	Unaddressed mental illness	~1 social worker per 75 patients (ref [11]; NKF/CNSW recommendation)
Preexisting psychiatric illness/trauma	Vulnerability to decompensation	Chronic depression, suicide risk	~20% of dialysis patients (ref [17])
COVID‐19 pandemic‐related stressors	Social isolation, disrupted routines—anticipated to worsen mood	No observed deterioration in Dutch dialysis cohort; possible protective effect of prior adaptation	Mental health scores unchanged vs. pre‐pandemic in Dutch DOMESTICO cohort (*n* = 177); authors attributed resilience to prior adaptation to restricted social life; ref. [21]

Abbreviations: BBB, blood–brain barrier; BH, behavioral health; CKD, chronic kidney disease; CVD, cardiovascular disease; DM, diabetes mellitus; DOPPS, Dialysis Outcomes and Practice Patterns Study; IL‐6, interleukin‐6; MDD, major depressive disorder; MH, mental health; PEW, protein‐energy wasting; QoL, quality of life; TNF‐α, tumor necrosis factor alpha.

## 5 Impact of Depression on Clinical Outcomes

Depression is the most common psychiatric disorder among CKD and ESKD patients, with approximately 25% experiencing clinically significant depression on structured interview, and prevalence rising as CKD progresses [15]. Depression has a substantial impact on outcomes in patients with CKD, contributing to increased cardiovascular events, greater healthcare utilization, reduced quality of life, and higher all‐cause mortality. The relationship between depression and CKD is bidirectional—baseline depression is associated with a higher risk of developing CKD, while CKD itself increases the likelihood of developing depression.

Management strategies typically involve pharmacologic therapy, psychotherapy, or both. Approximately 50% of adults with CKD and comorbid depression are treated with antidepressants, primarily selective serotonin reuptake inhibitors (SSRIs). However, the limited dialysis‐relevant trial evidence does not demonstrate that antidepressant monotherapy reliably outperforms placebo or psychotherapy for depressive symptoms. The CAST trial in non‐dialysis‐dependent CKD found no significant benefit of sertraline over placebo [23], and the ASCEND trial in maintenance hemodialysis found that sertraline and CBT produced similar modest improvements with neither demonstrating superiority over the other [24]. Antidepressant use in CKD/ESKD has not been shown to reduce mortality or hospitalization [25, 26].

Reconciling these observations is important. Management of psychiatric illness is further complicated by the tendency of some ESKD patients to underreport or deny psychiatric care, which can lead to nonadherence to treatment regimens [7, 16]. Inadequately managed symptoms can impair motivation for self‐management, including dialysis attendance. These findings highlight the limitations of monotherapy and reinforce support for integrated and multimodal treatment approaches. The current review endorses combination treatment (pharmacotherapy plus CBT) for the primary goal of improving depressive symptoms, quality of life, and treatment adherence rather than for the purpose of reducing hard clinical endpoints such as mortality or hospitalization, for which dialysis‐specific evidence remains insufficient. In other words, existing trials suggest that antidepressants alone produce modest symptomatic benefit without a clear hard‐outcome signal, while CBT and combination therapy appear to produce the most reliable improvements in patient‐reported outcomes. Dialysis‐specific head‐to‐head trials comparing combination therapy with either modality alone remain a significant evidence gap that should be prioritized in future research.

## 6 Pharmacotherapy Considerations in ESKD

Antidepressants in ESKD must be chosen with attention to pharmacokinetic changes (altered protein binding, reduced renal clearance of parent drug and/or active metabolites), dialyzability, QTc prolongation, serotonergic and bleeding risk, and interactions with the dialysis medication regimen [25, 27].

### 6.1 Preferred Agents

Sertraline and escitalopram are generally regarded as first‐line SSRIs in advanced CKD and dialysis because of predictable metabolism, relatively modest QTc effect at standard doses, low dialyzability (high protein binding, large volume of distribution), and an acceptable drug‐interaction profile. Typical starting doses are sertraline 25 mg daily (titrated to 50–100 mg) and escitalopram 5 mg daily (titrated to 10 mg; doses > 10 mg in dialysis should be used cautiously due to QTc signal). Mirtazapine (7.5–15 mg at bedtime, titrated cautiously) may be useful in patients with prominent insomnia, anorexia, or weight loss but requires monitoring for sedation and metabolic effects [27, 28].

### 6.2 Agents to Use With Caution or Avoid

Citalopram carries a dose‐dependent QTc prolongation warning, and the FDA recommends a maximum of 20 mg/day in patients at increased risk, a threshold that effectively includes most dialysis patients [29]. Paroxetine is generally avoided because of strong anticholinergic effects, sedation, discontinuation syndrome, and CYP2D6 inhibition that complicates polypharmacy. Tricyclic antidepressants (TCAs) should generally be avoided for depression in ESKD because of anticholinergic burden, orthostatic hypotension, QTc prolongation, and risk of cardiac arrhythmia; when used at low doses for neuropathic pain, close cardiac monitoring is warranted. Venlafaxine and desvenlafaxine can increase blood pressure and have active renally cleared metabolites requiring dose reduction [27]. Bupropion and its metabolites accumulate in renal failure, lower the seizure threshold, and should be used only at reduced doses with caution [30]. Lithium is contraindicated in dialysis outside of specialized psychiatric management while MAOIs require psychiatric subspecialty oversight and are rarely used [27].

### 6.3 QTc And Cardiac Considerations

Dialysis patients have high baseline rates of left ventricular hypertrophy, electrolyte shifts (particularly potassium, calcium, and magnesium) across the dialysis cycle, and concurrent exposure to QT‐prolonging agents including ondansetron, methadone, macrolide and fluoroquinolone antibiotics, azole antifungals, and antiarrhythmics such as sotalol and amiodarone. Before initiating an SSRI, a baseline ECG is reasonable when patients are on additional QT‐prolonging medications, and repeat measurement should be considered after dose titration. Citalopram, escitalopram at higher doses, and TCAs are the antidepressants of greatest QTc concern in this setting [31].

### 6.4 Drug–Drug Interactions and Bleeding Risk

SSRIs and SNRIs increase the risk of upper gastrointestinal bleeding, a clinically meaningful consideration in dialysis patients who are frequently anticoagulated (warfarin, direct oral anticoagulants), receive antiplatelet therapy, and have uremic platelet dysfunction [32, 33]. Fluoxetine and paroxetine are strong CYP2D6 inhibitors and fluvoxamine is a strong CYP1A2 inhibitor; these interact with several cardiovascular and immunosuppressive agents used in ESKD and transplant populations. Serotonergic medications should be co‐prescribed cautiously with tramadol, linezolid, methylene blue, and other serotonergic agents because of the risk of serotonin syndrome. In transplant candidates and recipients, interactions with calcineurin inhibitors (tacrolimus, cyclosporine) must be reviewed before initiation [27].

### 6.5 Dialyzability and Timing

Most clinically used SSRIs and SNRIs are minimally dialyzable due to high protein binding and large volumes of distribution, so supplemental dosing after hemodialysis is not required. Dose timing can be flexibly aligned with patient preference; adherence is often improved when medication is taken at a consistent, non‐dialysis‐related cue (e.g., morning or bedtime) [28].

### 6.6 Efficacy Data in Dialysis

Two pivotal randomized trials inform pharmacotherapy decisions in advanced kidney disease. The CAST trial (Hedayati et al. JAMA) [23] randomized 201 patients with stage 3, 4, or 5 non‐dialysis‐dependent CKD and major depressive disorder to sertraline or placebo for 12 weeks; sertraline did not significantly improve depressive symptoms compared with placebo, raising important questions about antidepressant monotherapy in advanced CKD. The ASCEND trial (Mehrotra and colleagues, Annals of Internal Medicine 2019) [24] randomized 120 patients receiving maintenance hemodialysis to 12 weeks of facility‐delivered CBT versus sertraline in a head‐to‐head comparison; both interventions produced modest reductions in depressive symptoms, with sertraline yielding a slightly greater reduction in BDI‐II at 12 weeks but with more adverse events than CBT. Taken together, these findings suggest that antidepressant monotherapy in advanced CKD/ESKD offers, at best, modest symptomatic benefit; that CBT is a reasonable first‐line or adjunctive option in motivated patients with access to a trained provider; and that combination therapy may be appropriate in patients with moderate‐to‐severe symptoms, partial response, or treatment‐resistant depression. Pharmacotherapy in this population should be individualized, regularly reassessed, and integrated with non‐pharmacologic care wherever possible.

## 7 Rationale for Integrated Mental Health Care in Dialysis

Integrated care models that combine routine screening, psychotherapy, pharmacotherapy, and psychiatric consultation offer a promising framework for addressing depression and anxiety in dialysis settings. We hypothesize, rather than claim as established, that implementation of an integrated treatment model could reduce depression severity as measured by validated scales such as the PHQ‐9 and, through structured referral pathways, improve medication adherence, appointment attendance, and downstream outcomes. This hypothesis is supported by indirect evidence from non‐dialysis populations and limited direct evidence in dialysis; prospective dialysis‐specific outcome trials are needed.

Several studies from psychiatry and primary care have examined the effectiveness of combining pharmacotherapy and psychotherapy for depression and anxiety. Meta‐analyses indicate that combination therapy is associated with higher response and remission rates, better adherence, and reduced relapse risk compared with either treatment alone [34, 35, 36]. Parallel analyses of specific psychotherapy modalities, including CBT, interpersonal therapy, and behavioral activation, show greater effectiveness when combined with pharmacotherapy than when used as monotherapy for moderate‐to‐severe depression.

A combined approach therefore represents a reasonable framework for managing depression and anxiety in ESKD. It should be noted, however, that the specific screening frequency, psychotherapy cadence, and referral threshold described in Figure 1 are proposed as an expert‐opinion framework drawing on general psychiatric best practice and adapted to the dialysis setting; they are not yet supported by dialysis‐specific comparative‐effectiveness or cost‐effectiveness trials. For example, monthly PHQ‐9 screening aligns with collaborative care models used in primary care and oncology and with CMS monthly‐visit rhythm, but its superiority over other cadences has not been established in dialysis. Similarly, weekly CBT delivered individually or in groups is a pragmatic approximation of standard CBT protocols but has not been formally tested against lower‐intensity schedules in dialysis populations. These elements should be understood as a proposed care model rather than an evidence‐based standard.

**FIGURE 1:**
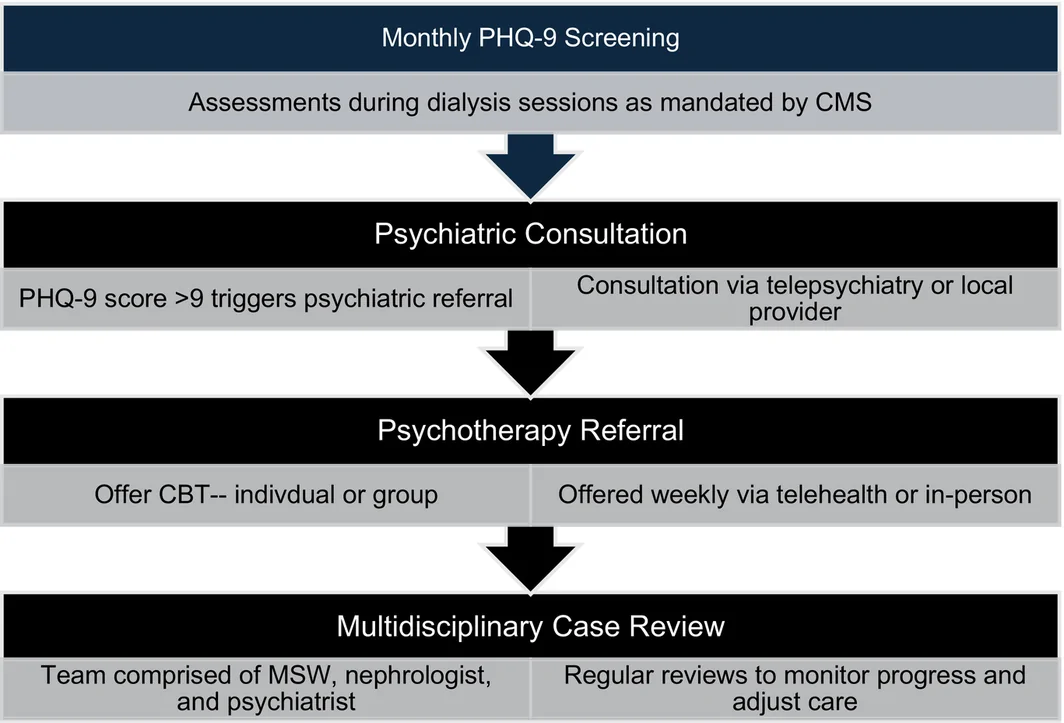
Proposed roadmap for behavioral health integration in dialysis clinics. This framework is proposed rather than evidence‐derived and should be adapted to local resources.

## 8 Effectiveness of CBT in Dialysis Patients

CBT has demonstrated modest but clinically meaningful improvements in depressive symptoms among patients with ESKD undergoing chronic hemodialysis. Several randomized controlled trials and non‐randomized studies support its efficacy in this population [6, 24, 37, 38, 39, 40, 41]. Lii and colleagues [37, 39] reported significant reductions in depressive symptoms and improvements in quality of life among Taiwanese hemodialysis patients, with benefits sustained over time. Duarte and colleagues [38, 40] reported improvements in depression and anxiety scores among Brazilian hemodialysis patients. A well‐powered RCT by Cukor et al. [6] demonstrated that CBT delivered by trained social workers significantly reduced depressive symptoms compared with usual care. Valsaraj and colleagues [39, 41] found that group‐based CBT improved both depression and coping strategies among Indian dialysis patients. In the United States, Lerma et al. [40] noted CBT‐associated improvements in mood, treatment adherence, and clinical outcomes. Griva et al. [41] showed that dialysis‐tailored CBT significantly reduced psychological distress and enhanced well‐being, with extension of benefits to caregivers. The multicenter ASCEND trial by Mehrotra et al. [24] confirmed the feasibility and effectiveness of CBT in real‐world dialysis settings, with statistically significant improvements in depression at 12 weeks; in head‐to‐head comparison with sertraline, CBT and pharmacotherapy produced similar modest benefit, neither demonstrating superiority.

In addition to RCTs, smaller uncontrolled or non‐randomized studies [42, 43, 44, 45] have demonstrated reductions in depressive symptoms following CBT or other psychological interventions. While CBT is generally considered effective and safe in kidney disease [46], limitations include restricted availability of trained providers, the self‐management burden placed on patients, and dialysis‐specific engagement barriers. Combination therapy integrating pharmacologic and psychotherapeutic interventions may be more effective than either alone in moderate‐to‐severe depression, potentially reducing relapse and recurrence [47, 48, 49].

## 9 Lessons Learned From Other Clinical Populations

Large clinical trials in primary care, oncology, and psychiatry underscore the effectiveness of collaborative care models in managing depression within medical settings. Many patients in these trials experience stressors and symptom constellations that overlap meaningfully with those faced by dialysis patients, making these models informative reference points. At the same time, direct extrapolation has important limitations that we discuss explicitly at the end of this section.

The STAR*D (Sequenced Treatment Alternatives to Relieve Depression) trial demonstrated that patients with depression present with similar symptom severity whether seen in psychiatric or general medical settings. Recurrent major depressive disorder was common in both groups but more prevalent among psychiatric patients (78% vs. 69%, *p* < 0.001), while chronic depression was more frequently observed in medical clinic populations (30% vs. 21%, *p* < 0.001). STAR*D emphasized the need for antidepressant trials of adequate dose and duration (≥ 8 weeks), highlighted that the probability of remission drops after two adequate trials, and recommended early referral to psychiatry or addition of cognitive therapy for patients with inadequate response [50].

The IMPACT (Improving Mood–Promoting Access to Collaborative Treatment) trial enrolled 1801 older adults with depression across multiple primary care settings. It evaluated a collaborative care model integrating depression care management, structured follow‐up, and psychiatric consultation into routine primary care. Patients receiving the IMPACT intervention were significantly more likely to experience a ≥ 50% reduction in depressive symptoms than those receiving usual care, and the intervention proved feasible in real‐world settings [51].

A comprehensive systematic review and meta‐analysis examining psychotherapy, antidepressant medication, and their combination in adults with major depressive disorder found that both psychotherapy alone and combination treatment were significantly more effective than pharmacotherapy alone for achieving remission and preventing relapse [52]. Research by Keller et al. [53] and Moret [54] further highlights that combination therapy produces faster remission, improved adherence, and greater overall effectiveness compared with monotherapy, particularly in moderate‐to‐severe depression.

Group psychotherapy has demonstrated robust efficacy across diverse populations and may be particularly well suited to dialysis patients who share common stressors and treatment experiences. A meta‐analysis by McDermut et al. [55] examined 48 studies of group psychotherapy for depression; 45 of the 48 reported significant symptom reductions, and group CBT was more effective than psychodynamic group therapy in the subset of studies making this comparison. Yalom and Leszcz [56] emphasized interpersonal learning as a core therapeutic mechanism in group therapy. Oncology models further suggest that group CBT can effectively reduce distress and enhance coping for both patients and caregivers [57, 58, 59].

### 9.1 Transferability to Dialysis: Limitations and Adaptations

Although these trials offer a strong conceptual foundation, their direct applicability to the dialysis population is limited, and this should be acknowledged rather than glossed over. First, STAR*D, IMPACT, and oncology CBT trials were conducted in ambulatory populations with lower comorbidity burden, no thrice‐weekly treatment schedule, and substantially lower symptom overlap between medical illness and depression than is encountered in ESKD. Second, dialysis patients face treatment‐specific fatigue and post‐dialysis “washout,” cognitive impairment from uremic toxin accumulation and intradialytic hemodynamic shifts, and transportation and scheduling constraints that reduce engagement with standard weekly outpatient therapy. Third, the reimbursement environment in US dialysis is fundamentally different: the ESRD Prospective Payment System (PPS) bundle constrains the financial viability of co‐located behavioral health services in ways not present in primary care or oncology. Fourth, group therapy benefits observed in oncology populations may not fully translate, as dialysis peer‐group dynamics involve visible treatment (fistulas, catheters, weight changes) and recurring shared clinical deterioration that may affect group cohesion differently. Elements most realistically adaptable to dialysis include the collaborative care infrastructure (registry‐based screening, care‐manager follow‐up, stepped‐care psychiatric consultation), the STAR*D emphasis on adequate antidepressant trial duration, and the empirical support for combination therapy. Elements requiring explicit dialysis‐specific adaptation include session timing (e.g., chair‐side brief CBT during dialysis), session length (shortened modules compatible with fatigue), provider type (appropriately trained MSWs and behavioral health clinicians), and payment model (CMS rule changes allowing separate billing for behavioral health within the ESRD bundle).

Dialysis‐specific evidence on group CBT, cost‐effectiveness of integrated care, and comparative effectiveness of different screening cadences remains a priority research gap.

## 10 Roadmap for Implementation in Dialysis Clinics (Figure 1)

Figure 1 presents a proposed, expert‐opinion‐derived framework for behavioral health integration in dialysis clinics, synthesizing elements from collaborative care models (IMPACT, STAR*D), dialysis‐specific CBT trials (ASCEND and others), and CMS regulatory requirements. Core components include: (1) routine depression screening with a validated instrument such as the PHQ‐9 at a cadence consistent with CMS requirements and clinical judgment; (2) stepped evaluation for patients with positive screens, including assessment by the dialysis social worker and referral for psychiatric evaluation when clinically indicated (e.g., PHQ‐9 ≥ 10, suicidality, functional decline, or failure to respond to initial management); (3) evidence‐based treatment with CBT (individual or group, adapted to dialysis constraints), pharmacotherapy selected with attention to renal considerations, or both; (4) structured follow‐up and measurement‐based care with periodic PHQ‐9 reassessment; and (5) team‐based integration among nephrology, nursing, social work, dietetics, and behavioral health. This model is proposed as a practical starting point; its specific components, cadence, and thresholds should be tailored to local resources and refined as dialysis‐specific evidence accumulates.

## 11 Limitations and Barriers Within the Dialysis Setting

Despite the conceptual promise of integrated care, several barriers hinder implementation. Patient‐level barriers include significant fatigue and lethargy during or after dialysis, which limit capacity for individual or group therapy; scheduling conflicts across thrice‐weekly dialysis sessions; stigma around psychiatric illness; and a tendency among some patients to minimize symptoms or resist establishing care with a psychiatric provider. Structural and clinical barriers include the scarcity of on‐site behavioral health providers, under‐recognition of psychiatric symptoms by nonmental‐health clinicians, and competing medical priorities.

Policy and payment barriers are particularly important. The ESRD Prospective Payment System (PPS) provides a single bundled payment to dialysis facilities that includes most renal‐dialysis‐related services and imposes consolidated billing, meaning that behavioral health services delivered within the bundle are not separately reimbursed even when provided by psychologists or psychiatrists [60]. Medicare Part B does cover outpatient mental health services, including those provided by psychologists or psychiatrists, but dialysis units cannot bill separately for these services unless they are furnished under separate provider arrangements (e.g., an independent physician practice or psychiatric clinic) [3, 61, 62]. Consequently, the absence of separate billing and limited financial incentives under current CMS policy discourages integration of behavioral health services in dialysis clinics [3]. These issues are addressed in the CY 2025 ESRD PPS rule and in MedPAC's June 2023 Report on Medicare behavioral health services [60, 61, 62].

Finally, this review has methodologic limitations. As a narrative rather than systematic review, it is subject to selection bias in the studies cited and does not provide quantitative pooled estimates. Recommendations for integrated care are supported primarily by indirect evidence from non‐dialysis populations and a small number of dialysis‐specific trials; cost‐effectiveness data specific to dialysis are largely absent.

## 12 Conclusion

ESKD is associated with a substantial burden of mental health disorders, particularly depression and anxiety, driven by a complex interplay of biological, psychological, and social determinants. Despite the high prevalence of mental illness in this population, integration of psychiatric care into dialysis settings remains inconsistent. Contributors to this gap include structural barriers such as the ESRD PPS reimbursement model, staffing constraints, and patient‐level factors including stigma, fatigue, and logistical challenges related to treatment schedules.

Evidence supports the efficacy of CBT in reducing depressive symptoms and improving adherence and quality of life in ESKD patients. Combination therapy integrating pharmacotherapy with CBT has demonstrated superior outcomes compared with monotherapy for moderate‐to‐severe depression in broader psychiatric populations, though dialysis‐specific comparative trials remain limited. Pharmacotherapy choices in ESKD require careful attention to dosing, dialyzability, QTc risk, and drug–drug interactions, with sertraline and escitalopram (at appropriately limited doses) generally favored over citalopram, paroxetine, tricyclics, and bupropion.

To address persistent gaps, a shift toward integrated, multidisciplinary care models is essential. This includes routine depression screening with validated tools such as the PHQ‐9, timely psychiatric referral, and structured delivery of CBT through individual or group formats adapted to the dialysis setting. Policy changes that permit reimbursement for behavioral health services in dialysis clinics, alongside investments in social work and psychiatric infrastructure, are critical to enabling this shift. Future research should prioritize dialysis‐specific randomized trials of collaborative care, comparative effectiveness of pharmacotherapy versus combination therapy, cost‐effectiveness analyses, and evaluation of group CBT protocols adapted to the dialysis context. Integrating nephrology and mental health care through comprehensive treatment pathways has the potential to meaningfully improve clinical outcomes, reduce healthcare utilization, and enhance well‐being for individuals living with ESKD.

## Conflicts of Interest

Kunal Malhotra has acted as a speaker for CSL Vifor, Travere Therapeutics, and Vantive. He has also participated in the Medical Advisory Board meeting for Fresenius Medical Care. No grant or other funding. Garima Singh has no conflict to report. No grant or other funding. Jordan Fangue‐Nelon has no conflict to report. No grant or other funding.

## Data Availability

Data sharing not applicable to this article as no datasets were generated or analysed during the current study.
